# Efficacy and safety of drug-eluting stents versus bare-metal stents in symptomatic intracranial and vertebral artery stenosis: a meta-analysis

**DOI:** 10.3389/fneur.2024.1389254

**Published:** 2024-11-05

**Authors:** Yidan Zhang, Wenbin Li, Lei Zhang

**Affiliations:** ^1^Department of Emergency, The Fifth Affiliated Hospital of Sun Yat-sen University, Zhuhai, China; ^2^Department of Neurovascular Disease, The Fifth Affiliated Hospital of Sun Yat-sen University, Zhuhai, China

**Keywords:** drug-eluting stent, bare-metal stent, intracranial artery stenosis, vertebral artery stenosis, meta-analysis

## Abstract

**Objectives:**

This study aims to present the first comprehensive meta-analysis assessing the effectiveness and safety of drug-eluting stents (DES) versus bare-metal stents (BMS) in treating intracranial and vertebral artery stenosis.

**Methods:**

A comprehensive examination was undertaken to compare the effectiveness and safety of DES and BMS in individuals experiencing symptomatic stenosis in the intracranial and vertebral arteries through an in-depth analysis of clinical research. We conducted an extensive search across multiple databases including PubMed, Embase, Web of Science, and the Cochrane Library up to September 2024. The emphasis of our investigation was on various outcomes including rates of in-stent restenosis, symptomatic occurrences of in-stent restenosis, incidence of stroke, procedural success, mortality rates, complications associated with the procedure, and any adverse events.

**Results:**

Our analysis included 12 studies with a total of 1,243 patients (562 in the DES group and 681 in the BMS group). The findings demonstrated a significantly lower rate of in-stent restenosis in the DES group for both intracranial [odds ratio (OR): 0.23; 95% confidence interval (CI): 0.13 to 0.41; *p* < 0.00001] and vertebral artery stenosis (OR: 0.38; 95% CI: 0.20 to 0.72; *p* = 0.003) compared to the BMS group. Additionally, the DES group showed a significantly reduced rate of postoperative strokes in vertebral artery stenosis cases (OR: 0.38; 95% CI: 0.16 to 0.90; *p* = 0.03), with no significant differences noted in the intracranial artery stenosis comparison (OR: 0.63; 95% CI: 0.20 to 1.95; *p* = 0.42). The study also revealed no significant disparities in symptomatic in-stent restenosis, procedural success, mortality, adverse effects, and perioperative complications between the two groups across the conditions studied.

**Conclusion:**

The comparison indicates that DES significantly reduces the risk of in-stent restenosis and postoperative strokes in patients with vertebral artery stenosis, compared to BMS. For both intracranial and vertebral artery stenosis, DES and BMS exhibit comparable safety profiles.

**Systematic review registration:**

https://www.crd.york.ac.uk/PROSPERO/display_record.php?RecordID=439967.

## 1 Introduction

Strokes’ prevalence globally is significantly impacted by intracranial artery stenosis, with 8 to 10% of strokes in North America and a substantial 30 to 50% in Asia being attributed to it (1–5). Responsible for 15 to 20% of posterior circulation strokes is vertebral artery stenosis (6, 7). Not only does this condition impede cerebral blood flow, leading to cerebral hypoperfusion, but it also serves as a potential source of arterial embolisms. Evidence from several studies suggests a link between vertebral artery stenosis and an increased likelihood of recurrent strokes (6, 8–10). Aggressive medical management is recommended as the cornerstone of stroke prevention for patients with intracranial and vertebral artery stenosis according to current guidelines (11). In substantially reducing the recurrence of strokes in these patients, this approach has been effective (2, 12, 13). Despite aggressive medical therapy, some patients still face a high risk of stroke recurrence (14, 15). When aggressive medical therapy fails, surgery and endovascular therapy become reasonable options. Due to the unique location of the vertebral and intracranial arteries, surgical revascularization is often challenging, with relevant perioperative complications and mortality related (16, 17).

Intravascular stents coated with anti-vascular endotheliocytes proliferation drugs on the surface or inside are known as drug-eluting stents (DES) (18). The sustained release of drugs on the surface of DES, compared with bare metal stents (BMS), can restrict the proliferation and migration of vascular smooth muscle cells in the stent, thereby restraining intravascular thrombosis and preventing in-stent restenosis. Mainly applied currently is DES to treat symptomatic stenosis in intracranial and vertebral arteries. Wu et al. (19) reported in a meta-analysis that drug-coated balloon angioplasty is a safe and effective method for the treatment of vertebral artery stenosis. Additionally, clinical studies have found that DES have a lower rate of restenosis for the treatment of intracranial and vertebral artery stenosis compared to BMS (18, 20–23). At present, the superiority of DES over BMS remains inconclusive due to factors such as the small sample size, short follow-up time, and variations in arterial stenosis sites. The first meta-analysis comparing the efficacy and safety of DES and BMS for intracranial and vertebral artery stenosis was reported.

## 2 Methods

### 2.1 Literature search

According to the PRISMA (Preferred Reporting Items for Systematic Reviews and Meta-Analysis) 2020 statement (24) and has been prospectively registered in the PROSPERO (CRD42023439967). Until September 2024, we meticulously searched through PubMed, Embase, Web of Science, and Cochrane databases to identify clinical studies comparing the efficacy and safety of DES with BMS in individuals suffering from symptomatic stenosis in intracranial and vertebral arteries. Through the following terms, we searched the literature extensively: “drug-eluting stents,” “bare-metal stent,” “intracranial,” “vertebral,” and “stenosis.” The detailed search strategies are as follows: (((“Drug-Eluting Stents”[Mesh]) OR (((((((((((((Drug Eluting Stents) OR (Stents, Drug-Eluting)) OR (Stents, Drug Eluting)) OR (Drug-Eluting Stent)) OR (Drug Eluting Stent)) OR (Stent, Drug-Eluting)) OR (Drug-Coated Stents)) OR (Drug Coated Stents)) OR (Stents, Drug-Coated)) OR (Stents, Drug Coated)) OR (Drug-Coated Stent)) OR (Drug Coated Stent)) OR (Stent, Drug-Coated))) AND ((bare-metal stent) OR (bare metal stent))) AND (((intracranial) OR (vertebral)) AND (stenosis)). Furthermore, we manually screened the bibliography lists of all included RCTs. Two authors independently gathered eligible articles and evaluated them. Any discrepancies in literature retrieval were resolved through discussion.

### 2.2 Inclusion and exclusion criteria

Eligible articles had to meet the following criteria:

P: patients diagnosed with symptomatic intracranial and vertebral artery stenosis.

I: DES.

C: BMS.

O: at least one outcome (such as in-stent restenosis, symptomatic in-stent restenosis, stroke, technical success, mortality, periprocedural complications, and adverse events) was measured; (5) data were available to analyze either odds ratios (OR) or weighted mean differences (WMD).

S: randomized controlled trial (RCT), cohort study, or case-control study.

Exclusions were made for study protocols, unpublished research, non-original studies (including letters, comments, abstracts, corrections, and replies), studies lacking adequate data, and review articles.

### 2.3 Data abstraction

Two authors independently conducted data abstraction, with any discrepancies resolved by a third author. We abstracted following information from eligible studies: first author name, published year, research period, study region, study design, intervention/exposure, control/non-exposure, sample size, age, gender, follow-up time, body mass index (BMI), comorbidity, stent length, in-stent restenosis, symptomatic in-stent restenosis, stroke, technical success, mortality, periprocedural complications, and adverse events. If the continuous data in the article was presented as median plus range or median plus interquartile range (IQR), we reanalysed the mean ± standard deviation (SD) via the methods reported by Wan et al. (25) and Luo et al. (26). Corresponding authors were contacted for full data if available, in cases where the research data is insufficient.

### 2.4 Quality evaluation

For evaluating the quality of eligible cohort studies, the Newcastle–Ottawa Scale (NOS) was utilized (27), with high quality being attributed to studies scoring 7–9 points (28). The quality assessment of eligible RCTs was conducted following the Cochrane Handbook for Systematic Reviews of Interventions 5.1.0 based on seven terms: random sequence generation, allocation concealment, blinding of participants and personnel, blinding of outcome assessment, incomplete outcome data, selective reporting, and other sources of bias (29). Every study aspect was assigned one of three evaluation outcomes: low risk, high risk, or unclear risk. Higher quality was attributed to studies with more evaluations indicating “low risk” bias. The quality of all included studies was independently assessed by two authors, with any disagreements settled through discussion.

### 2.5 Statistical analysis

The analysis utilized Review Manager 5.4.1 edition. Continuous data were synthesized using the WMD, while dichotomous data synthesis employed OR. Each metric was accompanied by 95% confidence intervals (CIs). To evaluate the heterogeneity of each outcome, the Cochran’s *Q* test (chi-squared test, *χ*^2^) and the inconsistency index (*I*^2^) were applied (30). *χ*^2^ *p*-value less than 0.1 or *I*^2^ more than 50% were regarded as high heterogeneity. The total WMD or OR for outcomes with significant heterogeneity (*χ*^2^ *p*-value less than 0.1 or *I*^2^ more than 50%) was calculated using the random-effects model. Otherwise, the fixed-effects model was applied. Additionally, subgroup analyses were conducted for outcomes with two or more included studies to evaluate possible confounders, if data were sufficient. Furthermore, we performed sensitivity analyses to evaluate the impact of each individual study on the overall WMD or OR when there were more than two studies included. Additionally, potential publication bias was assessed by generating funnel plots using Review Manager 5.4.1 and conducting Egger’s regression tests using Stata 15.1 edition (Stata Corp, College Station, Texas, United States) for outcomes involving more than two studies. A *p*-value less than 0.05 was deemed indicative of statistically significant publication bias.

## 3 Results

### 3.1 Literature retrieval, study characteristics, and baseline

**Figure 1:**
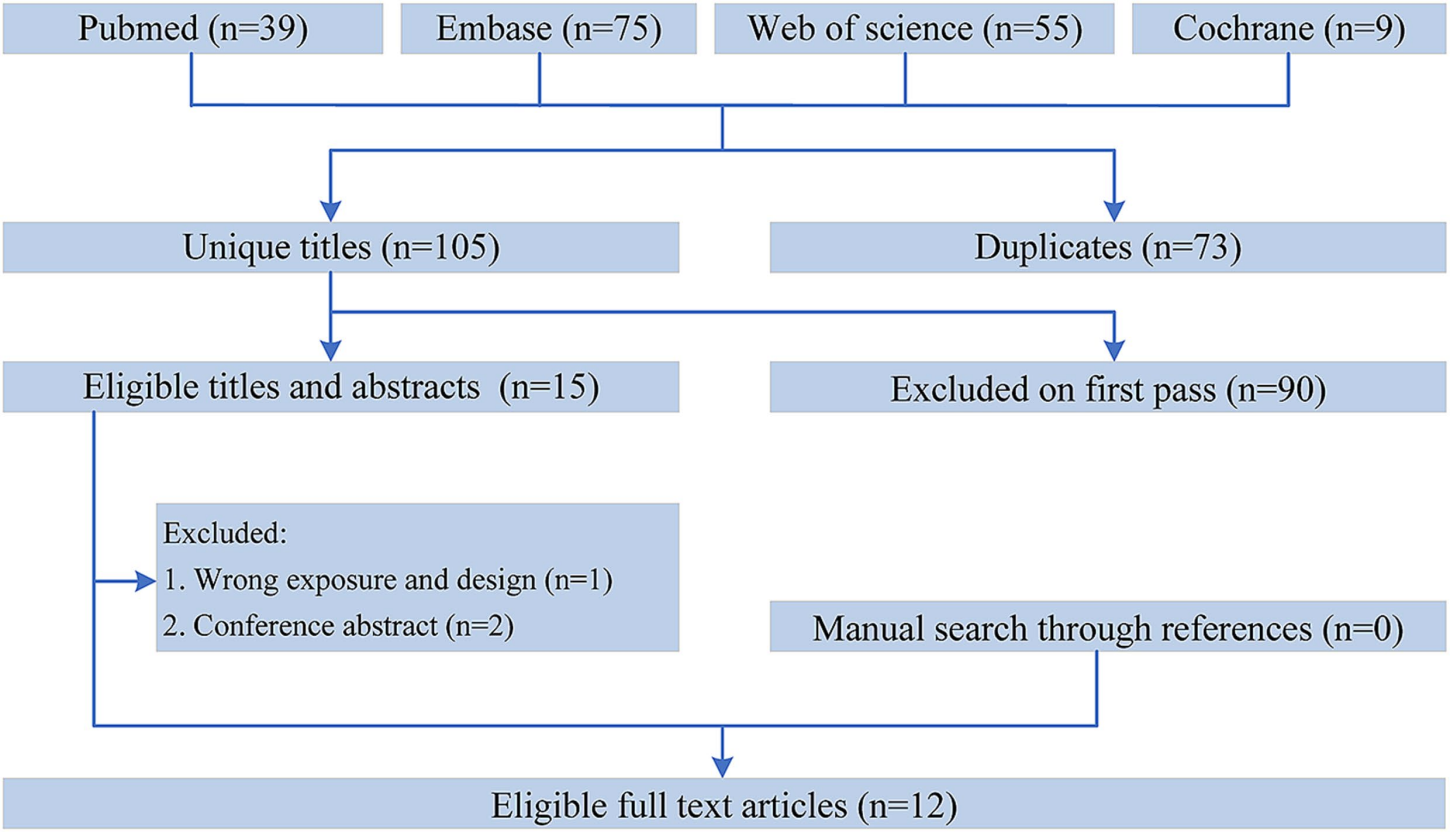
Flowchart of the systematic search and selection process.

**Figure 2:**
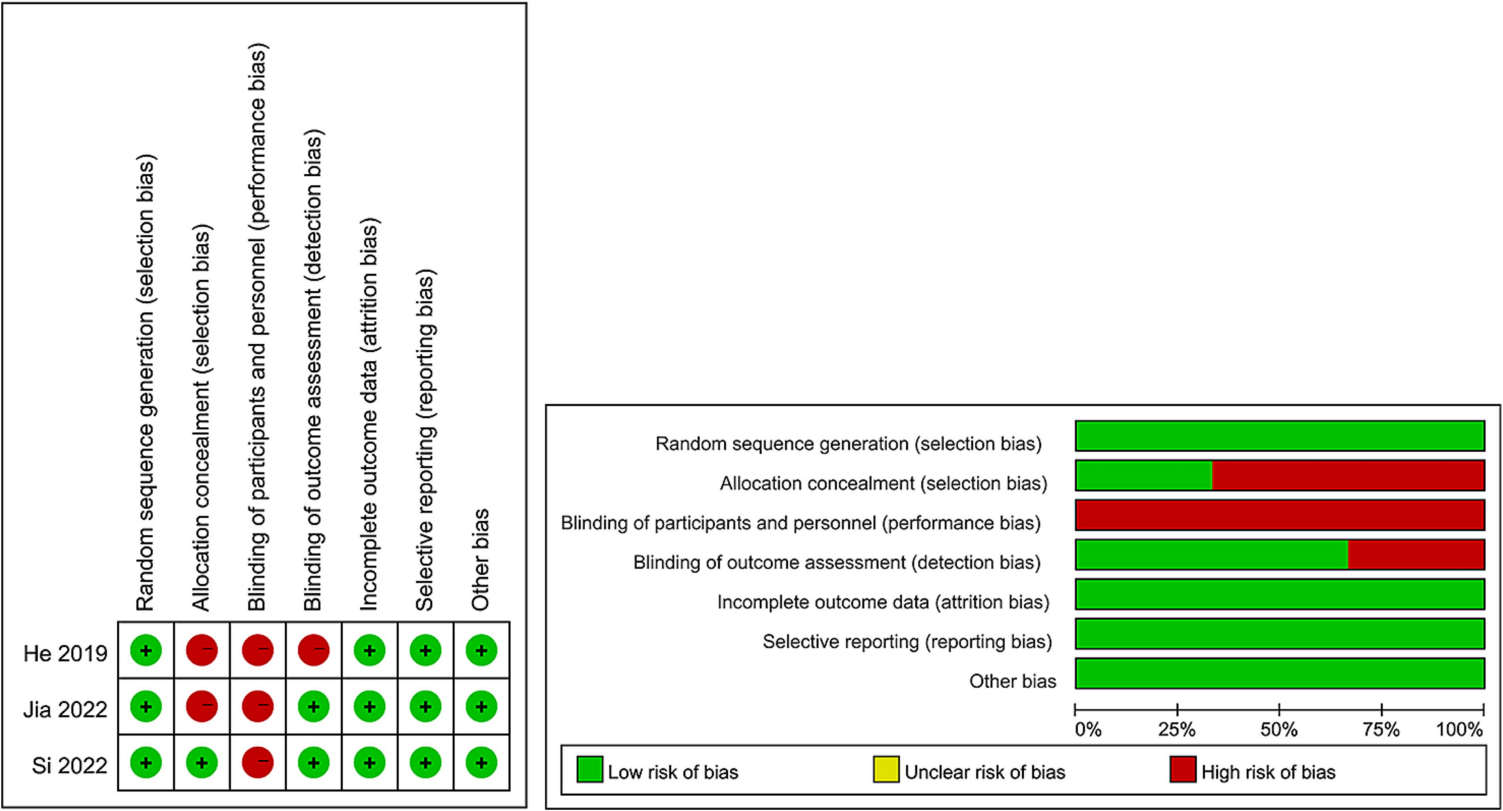
Details of the quality evaluation for included RCTs.

The flowchart of the literature retrieval and selection process is illustrated in Figure 1. Through systematic literature searches, a total of 178 related studies were identified in PubMed (*n* = 39), Embase (*n* = 75), Web of Science (*n* = 55), and Cochrane (*n* = 9). The detailed search strategy is presented in Supplementary Table S1. After the elimination of duplicate studies, 105 titles and abstracts underwent evaluation. Eventually, a meta-analysis was conducted, encompassing 12 studies and involving 1,243 patients (562 in the DES group versus 681 in the BMS group) (18, 20–23, 31–38). Presented in Table 1 are the characteristics and quality assessment of each eligible study. The details of quality evaluation for all included RCTs are shown in Figure 2, and Supplementary Table S2 displays the details of quality evaluation for all included cohorts. For intracranial artery stenosis, the two groups were comparable in age (WMD: 0.67; 95% CI: −1.04, 2.38; *p* = 0.44), gender (male) (OR: 1.01; 95% CI: 0.65, 1.57; *p* = 0.96), proportion of hypertension (OR: 0.74; 95% CI: 0.39, 1.41; *p* = 0.36), and proportion of coronary artery disease (OR: 1.03; 95% CI: 0.60, 1.77; *p* = 0.92) (Table 2). For cases of vertebral artery stenosis, both groups showed similar distributions in terms of gender (male) (OR: 0.92; 95% CI: 0.66, 1.26; *p* = 0.59), prevalence of hypertension (OR: 0.77; 95% CI: 0.36, 1.67; *p* = 0.51), prevalence of diabetes mellitus (OR: 1.05; 95% CI: 0.79, 1.40; *p* = 0.74), prevalence of coronary artery disease (OR: 1.30; 95% CI: 0.98, 1.74; *p* = 0.07), stent length (WMD: −0.25; 95% CI: −0.57, 0.08; *p* = 0.14), and BMI (WMD: −0.04; 95% CI: −1.14, 1.06; *p* = 0.94). The age of the DES group, however, was significantly lower than that of the BMS group (WMD: −1.29; 95% CI: −2.50, −0.09; *p* = 0.04) (Table 3).

**Table 1:** Baseline characteristics of include studies and methodological assessment.

Authors	Study period	Country	Study design	Location of stenosis	Intervention/exposure	Control/non-exposure	Stent implantation device	Preprocedural medical management	Post-procedure medical treatments	Patients (*n*)	Follow-up (mean/median)	Quality score
										DES/BMS		
Akins 2008	1999–2005	USA	Prospective cohort	Vertebral artery	Tacrolimus-eluting stents	Bare-metal stents	NA	Antiplatelet regimen	Aspirin (325 mg/day) and clopidogrel (75 mg/day) were prescribed for 6 months following the procedure, with either aspirin or clopidogrel indefinitely thereafter	5/7	30 months	6
Che 2018	2010–2016	China	Retrospective cohort	Vertebral artery	Drug eluting stent	Balloon-expandable bare-metal stent	6F or 8F guiding catheter (Mach 1; Boston Scientific, Marlborough, Mass)	Aspirin (100 mg/day) and clopidogrel (75 mg/day) for at least 2 days	Dual antiplatelet therapy	147/165	2.9 years	8
He 2019	2014–2015	China	RCT	Vertebral artery	Drug-eluting stent (Maurora stent)	Bare-metal stent (Apollo stent)	NA	NA	NA	20/20	18 months	—
Jia 2022	2015–2018	China	RCT	Intracranial arterial stenosis	Drug-eluting stent (NOVA intracranial sirolimus eluting stent system; SINOMED)	Bare-metal stent (Apollo intracranial stent system; MicroPort NeuroTech)	NA	Oral aspirin, 100 mg, daily and clopidogrel bisulfate, 75 mg, daily for at least 5 days before the procedure or a 300 mg loading dose of clopidogrel and a 100- to 300 mg loading dose of aspirin between 6 and 24 h before the procedure	Aspirin, 100 mg, daily and clopidogrel, 75 mg, daily for 90 days before converting to aspirin or clopidogrel alone	132/131	1 year	—
Langwieser 2014	1997–2012	Germany	Retrospective cohort	Vertebral artery	Balloon-expandable drug-eluting	Bare-metal stent (self-expanding and balloon-expandable)	An appropriate-shaped 6- or 7-French guiding catheter. The lesion was crossed with a 0.014-inch guide wire	Acetyl-salicylic acid (100–300 mg/day) and one additional platelet inhibitor including ticlopidin (500 mg TID as loading dose followed by 250 mg bid), clopidogrel (600 mg loading dose followed by 75 mg/day)	Thienopyridines were prescribed for at least 4 weeks or 6 months after bare-metal stent or drug-eluting stent implantation, respectively, whereas acetyl-salicylic acid was recommended as a life-long therapy	16/24	18 months	6
Lee 2013	2007–2012	Korea	Retrospective cohort	Intracranial arterial stenosis	Drug-eluting stent	Bare-metal stent	A 6 or 7 French guiding catheter was inserted into the proximal internal cerebral artery (ICA) after a femoral puncture. A 0.014 inch (0.35 mm) wire was then introduced through the guiding catheter with or without use of a microcatheter	All patients undergoing stent-angioplasty were given dual antiplatelet therapy for at least three days before the procedure, which consisted of aspirin (100 mg–300 mg/day orally) and clopidogrel (75 mg/day orally)	Dual antiplatelet therapy was continued after the procedure and then switched to monotherapy after at least 12 weeks (aspirin 100 mg/day orally)	7/24	40.7 months	7
Li 2020	2014–2015	China	Prospective cohort	Vertebral artery	Drug-eluting stent [Xience V (Abbott, United States), Endeavor (Medtronic, Ireland) and Firebird (MicroPort Medical, China)]	Bare-metal stent [Blue (Cordis Corp, Netherlands) and Express (Boston Scientific, United States)]	A 6-F guiding catheter was advanced into the subclavian artery proximal to the VAO with a 0.035-inch micro guidewire and the lesion was crossed with a 0.014-inch micro guidewire	Aspirin (100 mg/day) and clopidogrel (75 mg/day) were prescribed for all patients at least 5 days prior to the procedure	Double antiplatelet regimen (aspirin 100 mg daily, clopidogrel 75 mg daily) for 12 months and 3 months respectively, followed by aspirin monotherapy indefinitely	76/74	12.3 months	9
Maciejewski 2019	2003–2016	Poland	Prospective cohort	Vertebral artery	Drug-eluting stent	Bare-metal stent	Over a 0.035-inch diagnostic wire a 6 Fr guiding catheter was advanced toward the VA ostium. Then the lesion was crossed with a 0.014 inch coronary guide wire	One day before the procedure, patients received a 300 mg loading dose of clopidogrel	Patients were on aspirin 75 mg o.d., which was continued indefinitely afterwards	144/270	45.4 months	8
Raghuram 2012	—	USA	Retrospective cohort	Vertebral artery	Drug-eluting stent	Bare-metal stent	NA	3–5 days of dual antiplatelet preparation with aspirin and clopidogrel	NA	13/15	26 months	7
Si 2022	2014–2015	China	RCT	Intracranial arterial stenosis	Drug-eluting stent [Apollo stent (MicroPort Medical, Shanghai, China)]	Bare-metal stent [Maurora stent (Alain Medical, Beijing, China)]	A 0.014-inch micro guide wire was selected to pass through the stenosis through the guide tube	Clopidogrel 75 mg and aspirin 300 mg were started 3–5 days before the operation	Aspirin 100–300 mg/day and clopidogrel 75 mg/day were taken orally until 3 months after the operation; clopidogrel was stopped after 3 months; and aspirin was reduced to 100 mg/day for long-term use	92/96	1 year	—
Song 2012	2003–2010	China	Prospective cohort	Vertebral artery	Drug-eluting stent sirolimus-eluting stents [39 Cypher (Cordis Corporation, Bridgewater, NJ, United States) and 34 Firebird (MicroPort Medical, Shanghai, China)] or paclitaxel-eluting stents [52 Taxus (Boston Scientific Corporation, Natick, MA, United States)]	Bare-metal stent [balloon-expandable stents (63 stainless steel and 38 cobalt chromium)]	Lesions were crossed with a 0.014-inch microwire and microcatheter	Aspirin (300 mg/day) and clopidogrel (75 mg/day) at least 3 days before the treatment	Aspirin (100 mg/day) and clopidogrel (75 mg/day) for 12 months; after that, aspirin (100 mg/day) alone was continued indefinitely	112/94	43 months	9
Wang 2022	2018–2021	China	Retrospective cohort	Vertebral artery	Drug-eluting stent (XIENCE, Abbott, Temecula, California, United States)	Bare-metal stent (Apollo; MicroPort, Shanghai, China)	An 8F or 6F sheath was placed in the femoral artery. An 8F or 6F guide catheter was advanced to the subclavian artery proximal to the VAO.	Dual antiplatelet therapy consisting of aspirin (100 mg, daily), cilostazol (100 mg twice daily), and clopidogrel (75 mg, daily) or ticagrelor (90 mg, twice daily)	Dual antiplatelet therapy regimen was continued for 3 months in patients receiving stenting with BMS or angioplasty with DCB, and for 12 months in those receiving DES implantation. Antiplatelet monotherapy was used indefinitely	29/12	14.1 months	7

DES, drug-eluting stent; BMS, bare metal stent.

**Table 2:** Demographics and clinical characteristics of included studies for intracranial artery stenosis.

Outcomes	Studies	No. of patients	WMD or OR	95% CI	*p*-value	Heterogeneity			
		DES/BMS				Chi^2^	df	*p*-value	*I*^2^ (%)
Age (years)	3	231/251	0.67	[−1.04, 2.38]	0.44	0.30	2	0.86	0
Gender (male)	2	224/227	1.01	[0.65, 1.57]	0.96	0.05	1	0.83	0
Hypertension	2	224/227	0.74	[0.39, 1.41]	0.36	0.12	1	0.13	55
Coronary artery disease	2	224/227	1.03	[0.60, 1.77]	0.92	1.51	1	0.22	34

DES, drug-eluting stent; BMS, bare metal stent; WMD, weighted mean difference; OR, odds ratio; CI, confidence interval.

**Table 3:** Demographics and clinical characteristics of included studies for vertebral artery stenosis.

Outcomes	Studies	No. of patients	WMD or OR	95% CI	*p*-value	Heterogeneity			
		DES/BMS				Chi^2^	df	*p*-value	*I*^2^ (%)
Age (years)	6	394/485	−1.29	[−2.05, −0.09]	0.04^a^	8.89	5	0.11	44
Gender (male)	6	394/485	0.92	[0.66, 1.26]	0.59	2.67	5	0.75	0
Hypertension	7	399/492	0.77	[0.36, 1.67]	0.51	12.88	6	0.04	53
Diabetes mellitus	7	399/492	1.05	[0.79, 1.40]	0.74	5.81	6	0.44	0
Coronary artery disease	6	386/477	1.30	[0.98, 1.74]	0.07	2.66	5	0.75	0
Stent length	3	276/384	−0.25	[−0.57, 0.08]	0.14	1.17	2	0.56	0
BMI	2	49/32	−0.04	[−1.14, 1.06]	0.94	1.96	1	0.16	49

BMI, body mass index; DES, drug-eluting stent; BMS, bare metal stent; WMD, weighted mean difference; OR, odds ratio; CI, confidence interval.

a Statistically significant.

### 3.2 In-stent restenosis

**Figure 3:**
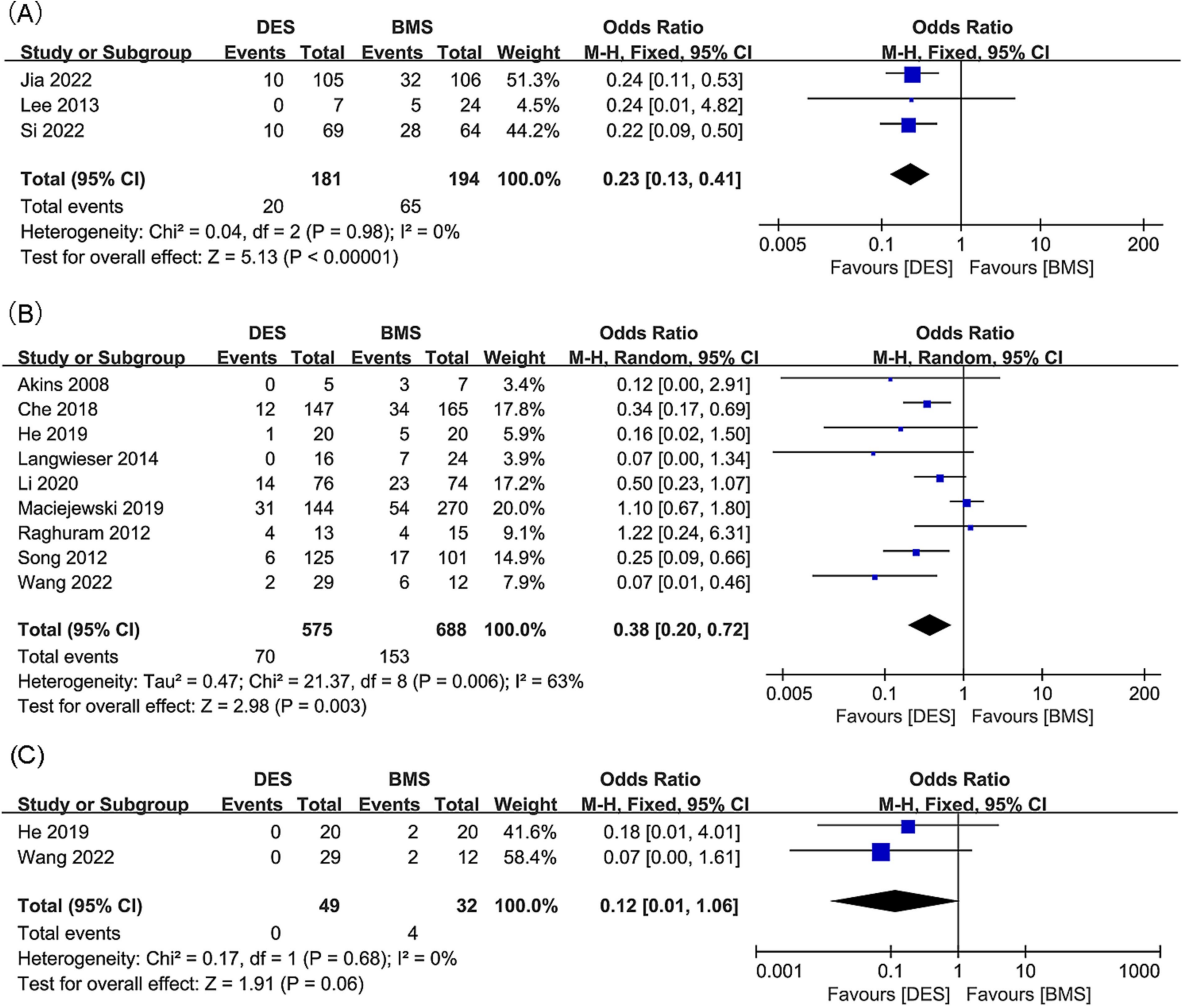
Forest plots of (A) in-stent restenosis (intracranial artery stenosis), (B) in-stent restenosis (vertebral artery stenosis), and (C) symptomatic in-stent restenosis (vertebral artery stenosis).

Intracranial artery stenosis, in-stent restenosis results were synthesized from 3 studies, including 375 patients (181 DES versus 194 BMS) (18, 21, 34). A significant lower in-stent restenosis rate in the DES group was revealed by meta-analysis (OR: 0.23; 95% CI: 0.13, 0.41; *p* < 0.00001), without significant heterogeneity observed (*I*^2^ = 0%, *p* = 0.98) (Figure 3A). Subgroup analysis unveiled that significance endured in prospective studies and those with a follow-up period of less than 24 months, yet dissipated in retrospective studies and those spanning 24 months or more in duration (Table 4).

**Table 4:** Subgroup analysis of DES versus BMS for intracranial arterial stenosis.

Subgroup	In-stent restenosis			
	Study	OR [95% CI]	*p*-value	*I* ^2^
Total	3	0.23 [0.13, 0.41]	<0.00001	0%
Study design				
Prospective	2	0.23 [0.13, 0.41]	<0.00001	0%
Retrospective	1	0.24 [0.01, 4.82]	0.35	NA
Follow-up				
≥24 months	1	0.24 [0.01, 4.82]	0.35	NA
<24 months	2	0.23 [0.13, 0.41]	<0.00001	0%

DES, drug-eluting stent; BMS, bare metal stent; OR, odds ratio; CI, confidence interval.

Findings regarding in-stent restenosis for vertebral artery stenosis were synthesized from 9 studies, which included 1,263 patients (575 DES versus 688 BMS) (20, 22, 23, 31–33, 35–37). A significant reduction in the rate of in-stent restenosis within the DES group was revealed by the meta-analysis (OR: 0.38; 95% CI: 0.20, 0.72; *p* = 0.003), along with significant heterogeneity (*I*^2^ = 63%, *p* = 0.006) (Figure 3B). Significance persisted in prospective studies, retrospective studies, and studies with a follow-up period <24 months, as well as in Asian studies according to subgroup analysis. However, it disappeared in studies with a follow-up period ≥24 months, as well as in European and American studies (Table 5).

**Table 5:** Subgroup analysis of DES versus BMS for vertebral arterial stenosis.

Subgroup	In-stent restenosis				Stroke				Periprocedural complications			
	Study	OR [95% CI]	*p*-value	*I* ^2^	Study	OR [95% CI]	*p*-value	*I* ^2^	Study	OR [95% CI]	*p*-value	*I* ^2^
Total	9	0.38 [0.20, 0.72]	0.003	63%	4	0.38 [0.16, 0.90]	0.03	0%	4	0.91 [0.41, 2.02]	0.82	0%
Study design												
Prospective	5	0.46 [0.21, 1.01]	0.05	64%	3	0.39 [0.15, 0.97]	0.04	0%	3	0.89 [0.39, 2.03]	0.78	0%
Retrospective	4	0.28 [0.09, 0.86]	0.03	51%	1	0.37 [0.05, 2.99]	0.35	NA	1	1.32 [0.05, 34.58]	0.87	NA
Follow-up												
≥24 months	5	0.51 [0.23, 1.15]	0.1	69%	2	0.42 [0.16, 1.12]	0.08	0%	2	0.62 [0.20, 1.88]	0.4	0%
<24 months	4	0.21 [0.07, 0.64]	0.007	43%	2	0.28 [0.05, 1.53]	0.14	0%	2	1.47 [0.44, 4.97]	0.53	0%
Region												
Asia	5	0.32 [0.20, 0.51]	<0.00001	10%	3	0.35 [0.13, 0.96]	0.04	0%	3	1.05 [0.41, 2.68]	0.91	0%
Europe	2	0.41 [0.03, 5.70]	0.51	70%	1	0.46 [0.10, 2.20]	0.33	NA	1	0.62 [0.12, 3.11]	0.56	NA
America	2	0.57 [0.06, 5.04]	0.61	40%								

DES, drug-eluting stent; BMS, bare metal stent; OR, odds ratio; CI, confidence interval.

### 3.3 Symptomatic in-stent restenosis

Two studies, including 81 patients (49 with DES and 32 with BMS), were conducted to synthesize data on symptomatic in-stent restenosis for vertebral artery stenosis (20, 32). No statistically significant difference was detected in the rate of symptomatic in-stent restenosis between the DES and BMS groups (OR: 0.12; 95% CI: 0.01, 1.06; *p* = 0.06), with no notable heterogeneity observed (*I*^2^ = 0%, *p* = 0.68) (Figure 3C).

### 3.4 Technical success

**Figure 4:**
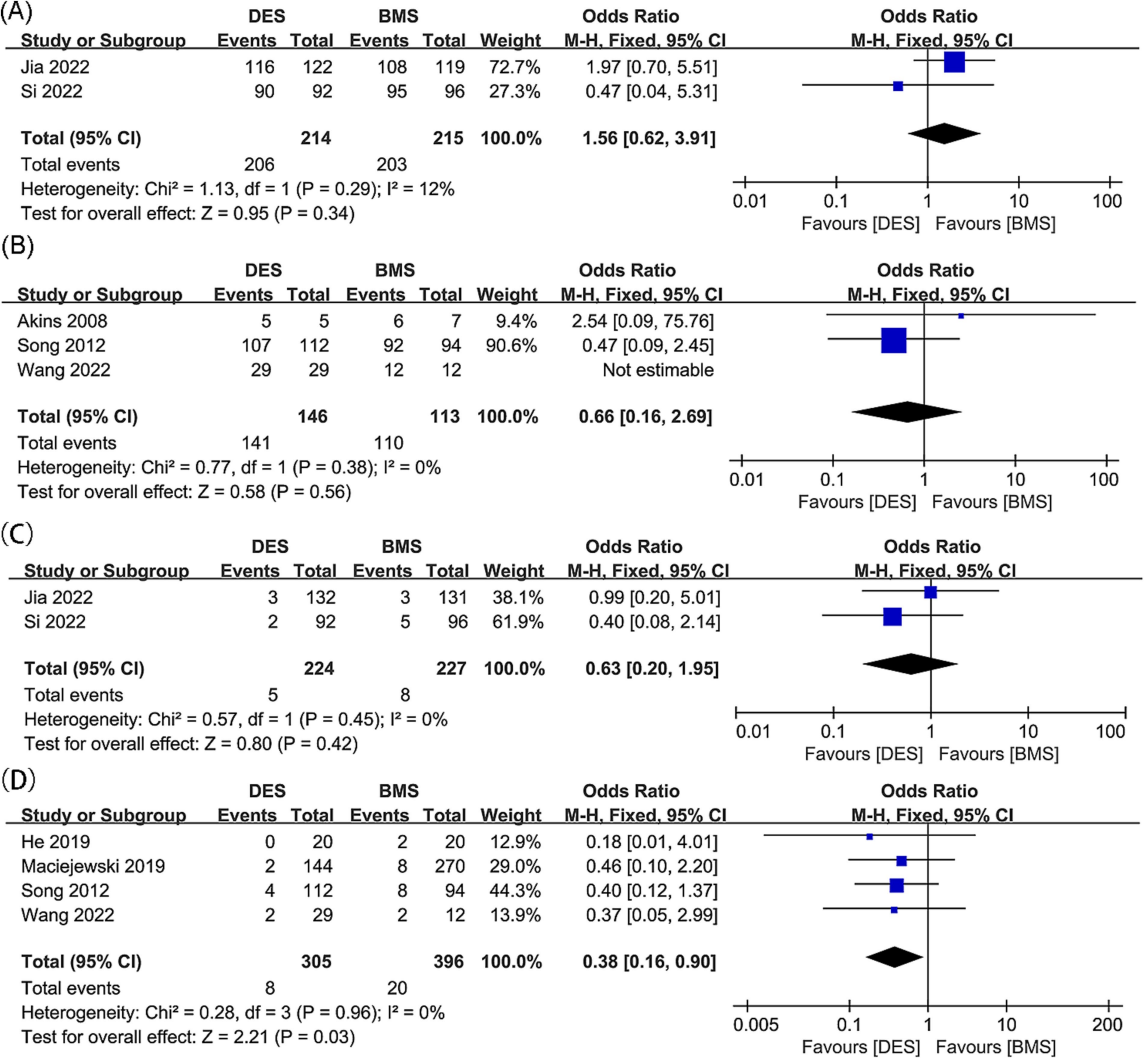
Forest plots of (A) technical success (intracranial artery stenosis), (B) technical success (vertebral artery stenosis), (C) postoperative stroke (intracranial artery stenosis), and (D) postoperative stroke (vertebral artery stenosis).

Results of technical success for intracranial artery stenosis were synthesized from two studies, which included 429 patients (214 treated with DES and 215 with BMS) (18, 21). No significant difference was found between the DES and BMS group for technical success rate, with an odds ratio of 1.56 (95% CI: 0.62, 3.91; *p* = 0.34). Additionally, no significant heterogeneity was observed (*I*^2^ = 12%, *p* = 0.29) (Figure 4A).

Technical success outcomes for vertebral artery stenosis were synthesized from three studies, with 259 patients included (146 DES versus 113 BMS) (20, 23, 31). No significant difference was found between the DES and BMS groups for technical success rate (OR: 0.66; 95% CI: 0.16, 2.69; *p* = 0.56), and no significant heterogeneity was observed (*I*^2^ = 0%, *p* = 0.38) (Figure 4B).

### 3.5 Postoperative stroke

Postoperative stroke outcomes for intracranial artery stenosis were collated from two studies, which included 451 patients (224 treated with DES versus 227 with BMS) (18, 21). There was no significant difference observed in the postoperative stroke rates between the DES and BMS groups (OR: 0.63; 95% CI: 0.20, 1.95; *p* = 0.42). Additionally, no significant heterogeneity was detected (*I*^2^ = 0%, *p* = 0.45) (Figure 4C).

No significant difference was found between the DES and BMS groups for postoperative stroke rate, with no significant heterogeneity observed (OR: 0.63; 95% CI: 0.20, 1.95; *p* = 0.42; *I*^2^ = 0%, *p* = 0.45) (20, 23, 32, 36). In the DES group, meta-analysis revealed a significant lower postoperative stroke rate (OR: 0.38; 95% CI: 0.16, 0.90; *p* = 0.03) without significant heterogeneity (*I*^2^ = 0%, *p* = 0.96) (Figure 4D). Subgroup analysis revealed that significance persisted in prospective and Asian studies, while it diminished in retrospective studies, as well as studies with follow-up periods of both less than 24 months and 24 months or more. Additionally, the significance was absent in European and American studies (Table 5).

### 3.6 Mortality

**Figure 5:**
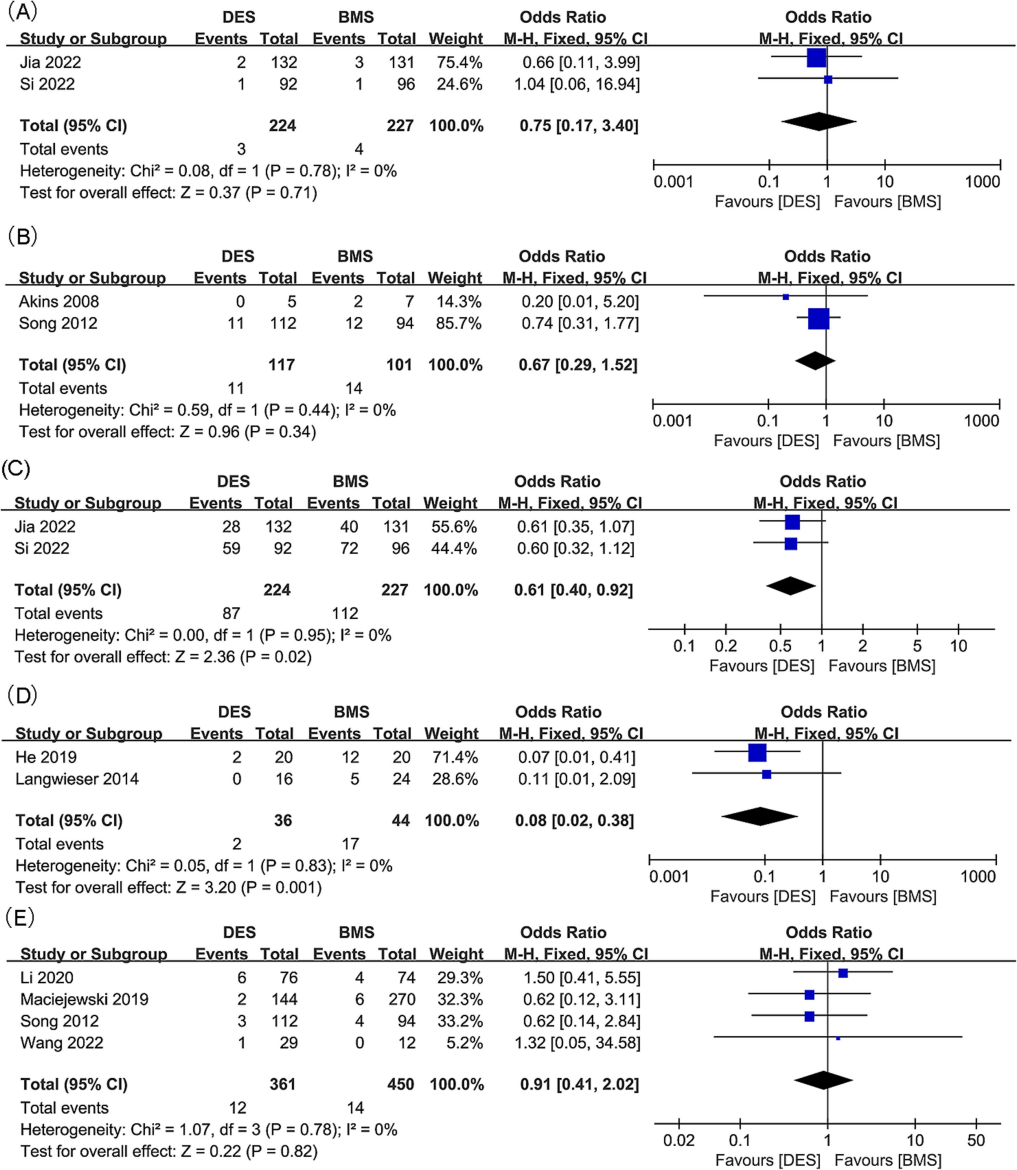
Forest plots of (A) mortality (intracranial artery stenosis), (B) mortality (vertebral artery stenosis), (C) adverse events (intracranial artery stenosis), (D) adverse events (vertebral artery stenosis), and (E) periprocedural complications (vertebral artery stenosis).

Mortality outcomes for intracranial artery stenosis were synthesized from two studies, which included 451 patients (224 with DES versus 227 with BMS) (18, 21). No significant difference was found in mortality between the DES and BMS groups (OR: 0.75; 95% CI: 0.17, 3.40; *p* = 0.71), and no significant heterogeneity was observed (*I*^2^ = 0%, *p* = 0.78) (Figure 5A).

Results of mortality for vertebral artery stenosis were synthesized from 2 studies, which included 218 patients (117 DES versus 101 BMS) (23, 31). No significant difference in mortality rates between the DES and BMS groups was observed (OR: 0.67; 95% CI: 0.29, 1.52; *p* = 0.34), and there was no significant heterogeneity noted (*I*^2^ = 0%, *p* = 0.44) (Figure 5B).

### 3.7 Adverse events

Adverse event outcomes related to intracranial artery stenosis were synthesized from two studies, which included 451 patients (224 with DES versus 227 with BMS) (18, 21). No significant difference was found between the DES and BMS groups for adverse events (OR: 0.63; 95% CI: 0.20, 1.95; *p* = 0.42), and no significant heterogeneity (*I*^2^ = 0%, *p* = 0.45) was observed (Figure 5C).

Results of adverse events for vertebral artery stenosis were synthesized from two studies, which included 80 patients (36 receiving DES versus 44 receiving BMS) (32, 33). In the DES group, a meta-analysis revealed a significantly lower rate of adverse events (OR: 0.08; 95% CI: 0.02, 0.38; *p* = 0.001), with no significant heterogeneity detected (*I*^2^ = 0%, *p* = 0.83) (Figure 5D).

### 3.8 Periprocedural complications

Four studies, comprising 811 patients (361 with DES and 450 with BMS), were analyzed to synthesize data on periprocedural complications related to vertebral artery stenosis (20, 23, 35, 36). No significant difference in periprocedural complications rate was observed between the DES and BMS groups (OR: 0.91; 95% CI: 0.41, 2.02; *p* = 0.82), with no significant heterogeneity detected (*I*^2^ = 0%, *p* = 0.78) (Figure 5E). Within all subgroups, the results consistently showed insignificance in the subgroup analysis (Table 5).

### 3.9 Publication bias

**Figure 6:**
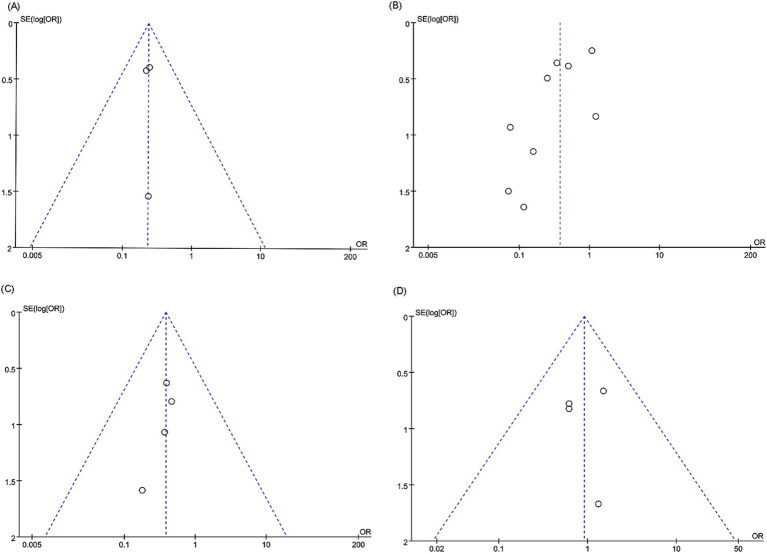
Funnel plots of (A) in-stent restenosis (intracranial artery stenosis), (B) in-stent restenosis (vertebral artery stenosis), (C) postoperative stroke (vertebral artery stenosis), and (D) periprocedural complications (vertebral artery stenosis).

For in-stent restenosis (intracranial artery stenosis), in-stent restenosis (vertebral artery stenosis), postoperative stroke (vertebral artery stenosis), and periprocedural complications (vertebral artery stenosis), we examined potential publication bias using funnel plots and Egger’s regression tests. Both statistical (Egger’s test) and visual (funnel plots) analyses detected no evidence of publication bias for in-stent restenosis in the intracranial artery (Egger’s test *p* = 1.000) (Figure 6A), in-stent restenosis in the vertebral artery (Egger’s test *p* = 0.050) (Figure 6B), postoperative stroke in the vertebral artery (Egger’s test *p* = 0.202) (Figure 6C), and periprocedural complications in the vertebral artery (Egger’s test *p* = 0.944) (Figure 6D).

### 3.10 Sensitivity analysis

**Figure 7:**
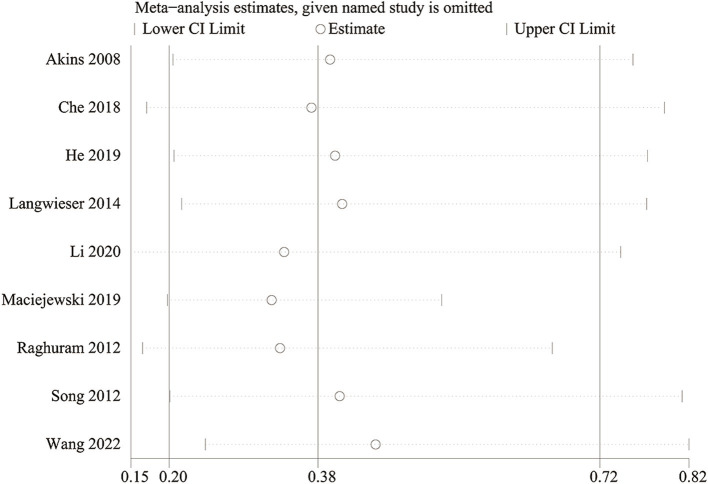
Sensitivity analysis of in-stent restenosis (vertebral artery stenosis).

We conducted a sensitivity analysis to evaluate the impact of each study on the overall OR for in-stent restenosis (specifically focusing on vertebra artery stenosis) by systematically excluding eligible studies one at a time. Our analysis revealed that even with the removal of each individual study, the total OR for in-stent restenosis (vertebral artery stenosis) remained consistent (Figure 7). However, upon removal of the study reported by Maciejewski et al. (36), the heterogeneity of in-stent restenosis (specifically, vertebral artery stenosis) decreased from 63 to 16%, suggesting that this paper might have been the main contributor to the significant heterogeneity observed in in-stent restenosis (vertebral artery stenosis).

## 4 Discussion

Stenting is considered inferior to aggressive medical therapy as a potential management strategy for intracranial or vertebral artery stenosis with impaired blood flow, primarily due to the high adverse event rates linked with stenting (13, 17, 39). In-stent restenosis presents another major obstacle to successful stenting, often resulting in non-procedural ischemic events (40, 41). In patients receiving the recent standard of self-expanding or balloon-installed BMS, in-stent restenosis appears within 1 year in 15 to 33% of cases (42–45). After stent implantation, DES reduce in-stent restenosis by restraining the proliferation and migration of endothelial cells and smooth muscle cells (46). In the treatment of arterial stenosis, DES has changed the status by reducing in-stent restenosis and associated ischemic events (47, 48). DES has been recommended as the standard device for percutaneous coronary intervention rather than BMS recently (49). However, the debate continues regarding whether DES outperforms BMS in terms of efficacy and safety for intracranial and vertebral artery stenosis.

To our knowledge, this is the first meta-analysis comparing DES and BMS for intracranial and vertebral artery stenosis. Despite differences in the design and type of stent used, similar results to some previous studies were shown in this study (18–21, 35). Among patients with stenosis in the intracranial and vertebral arteries, restenosis has been reported as a key factor affecting the long-term efficacy of treatment (20). Our study findings show a significant reduction in in-stent restenosis rates among patients with intracranial or vertebral artery stenosis who underwent treatment with DES compared to those who received BMS. The findings of our study are consistent with those of two previously published meta-analyses, which suggest that patients with symptomatic extracranial vertebral artery stenosis had a higher incidence of restenosis in the BMS group compared to DES (50, 51). Additionally, in two large case series, a higher risk of restenosis was found to be related to BMS in patients with vertebral artery stenosis (22, 52). The mechanism for the higher rate of restenosis in the BMS group is unclear. Studies have shown that the two main causes of restenosis in BMS may be recoil and intimal hyperplasia (53, 54). It is worth mentioning that, unlike DES, BMS does not apply antiproliferative medications that restrict smooth muscle cell proliferation. Therefore, it can be speculated that the deficiency of inhibition of intimal hyperplasia in the BMS group resulted in the most severe stenosis advancement and the highest restenosis rate during follow-up in the BMS group.

Furthermore, our meta-analysis found a significant lower postoperative stroke rate in the DES group for vertebral artery stenosis, while no significant difference was observed between the DES and BMS group for intracranial artery stenosis. Despite all four articles addressing postoperative stroke (vertebral artery stenosis) and reporting non-significant differences between DES and BMS, the outcomes of the studies were all skewed in favor of the DES group. After conducting pooled analysis, it was ultimately determined that the DES cohort exhibited a reduced incidence of postoperative stroke, with no notable heterogeneity observed as a result. This discovery could be attributed to the reduced incidence of in-stent stenosis observed in the DES cohort. Our study found no significant difference, generally, in mortality, adverse effects, and perioperative complications between the DES group and the BMS group. Despite the meta-analysis indicating a significant reduction in adverse events rates within the DES group compared to the BMS group for vertebral artery stenosis, it’s important to acknowledge that only two original studies contributed to this finding. Therefore, further research is essential to corroborate these results. In addition, subgroup analysis found that the advantage of DES in restenosis rate disappeared when the follow-up time exceeded 24 months. This finding may be related to the characteristics of DES. As time goes by, the dose of drugs released by DES will gradually decrease, and its average service life is 3–10 years, so its long-term treatment effect may not be significantly better than BMS (55). In addition, although DES can reduce the restenosis rate, long-term drug release and polymer stimulation may increase the risk of thrombosis, which will also affect the long-term efficacy (55).

We must acknowledge several limitations of this meta-analysis. Firstly, all included RCTs reported a high risk in the blinding of participants and personnel because of the deficiency of feasibility of blinding for the type of stents used, and only 1 of 3 included RCTs had low risk in the allocation concealment, which may lead to some bias. Secondly, most of the original studies on intracranial arterial stenosis included in this meta-analysis did not consider the separation of patients with anterior and posterior circulation. Due to the different natural history of arterial stenosis in the anterior and posterior circulation, the operation method, clinical prognosis and restenosis rate after stenting are also different. Thirdly, we conducted subgroup analysis on some outcomes according to the study design, follow-up time and region, and the subgroup analysis found that the results were not stable in different subgroups, suggesting that this study still had a certain degree of heterogeneity, although most the results are reported as non-significant Cochran’s *Q p*-values. In addition, the drug eluting inhibitors involved in this article mainly include tacrolimus, sirolimus and paclitaxel, which may be one of the sources of heterogeneity. However, there are few literatures reporting specific drug eluting inhibitors, and we cannot perform subgroup analysis based on this factor to explore its impact on the results. Besides, in all included literature, the most common pre- and post-implantation treatment drugs included aspirin, clopidogrel, and ticlopidin. However, because the patients included in each study had different disease courses and characteristics, the pre- and post-implantation drug doses and treatment courses were very heterogeneous among the studies, and subgroup analysis could not be performed, although we believe that different antiplatelet regimens will affect the treatment success rate and long-term prognosis of patients. Despite several limitations of this meta-analysis, we conducted the first meta-analysis comparing DES and BMS for intracranial and vertebral artery stenosis. Results of this meta-analysis validated the superiority of the DES intracranial and vertebral artery stenosis reported by previous studies.

## 5 Conclusion

Pooled analyses have revealed that DES, when compared to BMS, exhibit a significant reduction in the risk of in-stent restenosis (including intracranial and vertebral artery stenosis) as well as postoperative stroke (particularly in cases of vertebral artery stenosis). However, this superiority appears to be limited to a relatively short follow-up period. Generally, DES and BMS demonstrate similar safety profiles for intracranial and vertebral artery stenosis. To further evaluate the efficacy and safety of drug-eluting stents versus bare-metal stents in patients with symptomatic intracranial and vertebral artery stenosis, additional large-scale, multi-center, double-blind RCTs are warranted.

## Data Availability

The original contributions presented in the study are included in the article/Supplementary material, further inquiries can be directed to the corresponding author.
