# Supplementary material for: Efficacy and safety of drug-eluting stents versus bare-metal stents in symptomatic intracranial and vertebral artery stenosis: a meta-analysis
Source: Front Neurol. 2024 Nov 5;15:1389254. doi: 10.3389/fneur.2024.1389254 (PMC11573555; doi:10.3389/fneur.2024.1389254)
Supplement: Supplementary file 1 [file Table_1.DOCX]

Supplementary Material

**Supplementary Table 1.** The searching strategy

| 1. Pubmed   ((("Drug-Eluting Stents"[Mesh]) OR (((((((((((((Drug Eluting Stents) OR (Stents, Drug-Eluting)) OR (Stents, Drug Eluting)) OR (Drug-Eluting Stent)) OR (Drug Eluting Stent)) OR (Stent, Drug-Eluting)) OR (Drug-Coated Stents)) OR (Drug Coated Stents)) OR (Stents, Drug-Coated)) OR (Stents, Drug Coated)) OR (Drug-Coated Stent)) OR (Drug Coated Stent)) OR (Stent, Drug-Coated))) AND ((bare-metal stent) OR (bare metal stent))) AND (((intracranial) OR (vertebral)) AND (stenosis)) |
| --- |
| 1. Embase and Cochrane (via Ovid)   (Drug-Eluting Stents or (Drug Eluting Stents or Stents, Drug-Eluting or Stents, Drug Eluting or Drug-Eluting Stent or Drug Eluting Stentor Stent, Drug-Eluting or Drug-Coated Stents or Drug Coated Stents or Stents, Drug-Coated or Stents, Drug Coated or Drug-CoatedStent or Drug Coated Stent or Sent, Drug-Coated) and (bare-metal stent or bare metal stent) and ((intracranial or vertebral) andstenosis)).af. |
| 1. Web of Science   (((Drug-Eluting Stents) OR (((((((((((((Drug Eluting Stents) OR (Stents, Drug-Eluting)) OR (Stents, Drug Eluting)) OR (Drug-Eluting Stent)) OR (Drug Eluting Stent)) OR (Stent, Drug-Eluting)) OR (Drug-Coated Stents)) OR (Drug Coated Stents)) OR (Stents, Drug-Coated)) OR (Stents, Drug Coated)) OR (Drug-Coated Stent)) OR (Drug Coated Stent)) OR (Stent, Drug-Coated))) AND ((bare-metal stent) OR (bare metal stent))) AND (((intracranial) OR (vertebral)) AND (stenosis)) (Topic) and Preprint Citation Index (Exclude – Database) |

##

**Supplementary Table 2.** Quality evaluation of the eligible studies with Newcastle–Ottawa scale.

| Study | Selection | | | | Comparability | | Outcome | | |
| --- | --- | --- | --- | --- | --- | --- | --- | --- | --- |
|  | Representative-ness | Selection of  non-exposed | Ascertainment  of exposure | Outcome not present at start | Comparability on most important factors | Comparability on other risk factors | Assessment of outcome | Long enough follow-up (median≥1 year) | Adequacy  (completeness) of follow-up |
| Akins 2008 | 0 | 1 | 1 | 1 | 0 | 0 | 1 | 1 | 1 |
| Che 2018 | 1 | 1 | 1 | 1 | 1 | 0 | 1 | 1 | 1 |
| Langwieser 2014 | 0 | 1 | 1 | 1 | 0 | 1 | 1 | 1 | 0 |
| Lee 2013 | 0 | 1 | 1 | 1 | 0 | 1 | 1 | 1 | 1 |
| Li 2020 | 1 | 1 | 1 | 1 | 1 | 1 | 1 | 1 | 1 |
| Maciejewski 2019 | 1 | 1 | 1 | 1 | 1 | 0 | 1 | 1 | 1 |
| Raghuram 2012 | 0 | 1 | 1 | 1 | 0 | 1 | 1 | 1 | 1 |
| Song 2012 | 1 | 1 | 1 | 1 | 1 | 1 | 1 | 1 | 1 |
| Wang 2022 | 0 | 1 | 1 | 1 | 0 | 1 | 1 | 1 | 1 |
|  | | | | | | | | | |
